# Walking on a Vertically Oscillating Treadmill: Phase Synchronization and Gait Kinematics

**DOI:** 10.1371/journal.pone.0169924

**Published:** 2017-01-18

**Authors:** Jeff A. Nessler, Severne Heredia, Jacques Bélair, John Milton

**Affiliations:** 1 Department of Kinesiology, California State University, San Marcos, California, United States of America; 2 Department of Mathematics and Statistics, University of Montreal, Quebec, Canada; 3 W. M. Keck Science Center, Claremont College, Claremont, California, United States of America; Fondazione Santa Lucia Istituto di Ricovero e Cura a Carattere Scientifico, ITALY

## Abstract

Sensory motor synchronization can be used to alter gait behavior. This type of therapy may be useful in a rehabilitative setting, though several questions remain regarding the most effective way to promote and sustain synchronization. The purpose of this study was to describe a new technique for using synchronization to influence a person’s gait and to compare walking behavior under this paradigm with that of side by side walking. Thirty one subjects walked on a motorized treadmill that was placed on a platform that oscillated vertically at various frequencies and amplitudes. Synchronization with the platform and stride kinematics were recorded during these walking trials and compared with previously reported data from side by side walking. The results indicated that vertical oscillation of the treadmill surface at frequencies that matched subjects preferred stride or step frequency resulted in greater unintentional synchronization when compared with side by side walking data (up to 78.6±8.3% of the trial vs 59.2±17.4%). While intermittent phase locking was observed in all cases, periods of synchronization occurred more frequently and lasted longer while walking on the oscillating treadmill (mean length of periods of phase locking 11.85 steps vs 5.18 steps). Further, stride length, height and duration were altered by changing the frequency of treadmill oscillation. These results suggest that synchronization to a haptic signal may hold implications for use in a clinical setting.

## Introduction

The use of sensory motor synchronization to alter the control of gait appears promising for restoring certain aspects of locomotor control [1–4]. To date, applications in rehabilitative settings include stroke [1,5–7], Parkinson’s disease [2,4,8], Huntington’s disease [9], and traumatic brain injury [3]. The use of synchronization as a form of therapy is based upon the assumption that interactions between two oscillators are highly stereotyped and do not depend on the mechanisms that generate the oscillations. Hence, synchronization may arise between two very different oscillators, such as two people walking side by side [10–12] or a person responding to a periodic auditory cue provided by a metronome [1,5,13]. However, the best approach for implementing therapeutic interventions based on these effects remains to be determined.

In general, two strategies for gait synchronization have been studied. The first requires the individual to intentionally synchronize their gait to an acoustic, pacing stimulus [1,5–7,13]. This approach is a periodic forcing strategy since the acoustic rhythm affects the walker’s gait rhythm, but not vice versa. Currently, gait synchrony to a non-adapting auditory cue is not fully understood and may lead to walking dynamics that are different from those of natural, uncoupled human gait [14–16]. The second strategy involves synchronization of gait to that of another person walking at one’s side [10–12,17]. Since reciprocal interaction occurs between the two walkers, changes in gait are likely due to interactive coupling between the two gait rhythms [12,18,19]. Evidence suggests that subconscious, spontaneous synchronization promotes walking behavior that is closer to that of normal walking when compared to the intentional case [16,20]. However, without the intent to synchronize, side by side walking (SBSW) has a relatively weak coupling strength and hence the utility of SBSW to influence a person’s gait is limited [10,12,21].

Here a new strategy for gait synchronization is introduced that utilizes a periodic forcing strategy and increases the coupling strength between walker and external cue without requiring the intentional dependence of the walker on the stimulus. Specifically, an actuated platform was developed which vertically oscillates a treadmill together with a walking subject over a range of frequencies and amplitudes that can effectively alter gait dynamics. Previous studies suggest that haptic stimuli [10,11] and the “wobbly bridge” phenomenon [22] can increase the coupling strength between walker and the oscillating walking surface. The purpose of this study is to characterize the behavior of a healthy walking human in response to small amplitude, vertical platform oscillations (vertical oscillating treadmill walking, VOTW) and compare the effects on gait synchronization to those observed for SBSW.

## Methods

### Subjects

A convenience sample of 31 subjects was recruited from the local student population (age 24.7±3.9 years, mass 64.5±9.2 kg, height 1.7±0.1 m, leg length 78.5±4.9 cm). All subjects were free of any neurological or musculoskeletal impairment that may have affected gait and/or balance. All procedures were approved by the Institutional Review Board at California State University, San Marcos, and all participants gave their written informed consent prior to any data collection.

### Apparatus

**Fig 1:**
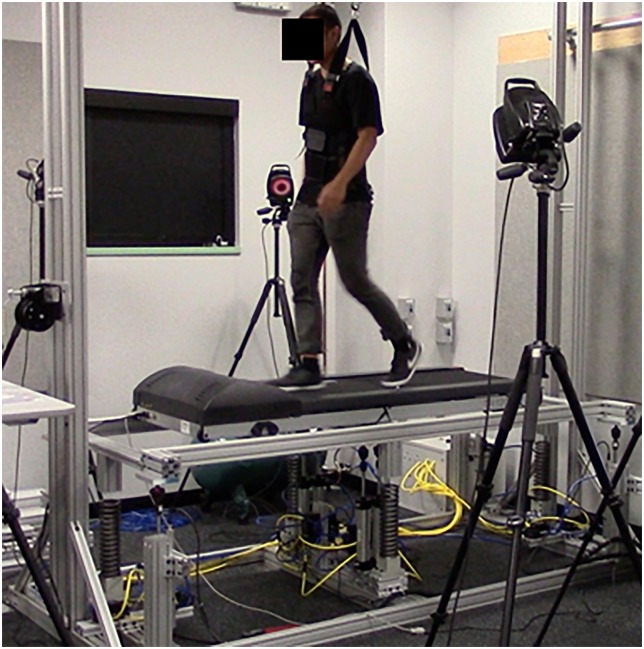
The oscillating platform and walking subject. A 6-camera motion capture system was used to acquire data, and an overhead safety harness was used to ensure subject safety.

Participants walked on a motorized treadmill that was placed on an elevated, moveable platform (Fig 1). This platform was actuated by 8 pneumatic cylinders (TRD Manufacturing, Machesney Park, IL): one beneath each corner of the platform, and 4 cylinders beneath the center. All cylinders were supplied with 655 kpa compressed air. The endpoint position of each cylinder was individually controlled by an electronic position control valve (Enfield Technologies, Shelton, CT) and custom software created in MATLAB’s xPC Target environment (R2014, Natick, MA). The platform could be made to oscillate vertically while a subject walked on the treadmill (maximum amplitude ≈ 15 cm; maximum frequency ≈ 3 Hz). An overhead gantry (custom built) and harness (Maine Anti-Gravity Systems, Inc.) were provided to ensure subject safety. A small amount of slack was maintained to ensure that subjects bore their entire body weight during all walking trials. While walking on the apparatus, subjects were not explicitly instructed to synchronize with the oscillations of the walking surface, but were instructed to walk as normally as possible. Exhaust from the pneumatic system was ported to a soundproof box in an effort to minimize audible cues that may have influenced the walking participant.

### Preferred Stride Frequency (f_0_)

Treadmill speed was held constant at 4.02 km/h and the treadmill oscillation amplitude, *A*, was set to 0cm. Each subject had a 2 min warm-up walking on the treadmill and their preferred stride frequency, *f*_*0*_, was determined from the next 2 min of treadmill walking.

### Gait Synchronization

Gait synchronization to the platform vertical oscillation frequency, *f*, was measured for 26 of the 31 subjects (age 24.2±3.3 years, mass 64.1±10.5kg, height 1.7±0.1 m, leg length 78.9±4.7 cm) in the following manner. Treadmill speed and desired platform oscillation amplitude, A, were held constant at 4.02 km/h and 2.5 cm, respectively. Platform oscillation height (i.e. amplitude) was relatively consistent across all trials (mean standard error = ±1.0mm), but the absolute error in platform position increased to as much as 10mm in a few instances when heavier subjects walked on the treadmill at higher frequencies. This was due primarily to the power limitations of the pneumatic system. Subjects also experienced small fluctuations in amplitude from stride to stride, and this variation was typically between 1 and 2mm. Though there was potential for treadmill amplitude to vary somewhat among participants due to their weight, amplitudes were highly consistent within subjects and across the different treadmill frequencies analyzed here. The effects of these amplitude changes on phase locking are analyzed in more detail below.

Each subject was studied on one day and completed a total of 10 walking trials, each lasting 2 min as follows: 2 min warm-up, 2 min to estimate their *f*_*0*_ when A = 0, and eight 2 min trials each with a different *f*. These eight trials were presented in random order in which *f* was varied as follows: 1) *f* = *f*_*0*_ (subject’s preferred stride frequency), 2) *f* = 2 × *f*_*0*_ (subject’s estimated step frequency), 3) *f* = 1.05 × *f*_*0*_, 4) *f* = 0.95 × *f*_*0*_, 5) *f* = 2.10 × *f*_*0*_, 6) *f* = 1.90 × *f*_*0*_, 7) *f* = 2.20 × *f*_*0*_, and 8) *f* = 1.80 × *f*_*0*_. The range of f is comparable to the average variation of subjects’ walking speed observed previously [23]. Detailed descriptions of the analysis of synchronization for side by side walking subjects have been discussed previously [10,21]. The values of ϕ were determined using a 6 camera motion capture system (Vicon, Oxford, UK) to track 4 reflective markers: 2 markers were placed on the lateral malleoli of each ankle and 2 were placed on the treadmill frame on either side of the treadmill belt. Relative phase (ϕ) was calculated according to the equation,

$$\Phi =Phase_{Treadmill}-Phase_{Subject}$$ (1)

Where:

$$Phase_{i,j}=\frac{2\times \pi \times i}{q_{j}}$$ (2)

Here, *j* represents the cycle number (treadmill or stride), *i* represents the data point within the respective cycle *j*, and q_j_ represents the total number of data points within the cycle *j*. Heel strike defined the beginning of each stride cycle, and each treadmill cycle began when the platform was at its lowest height. A relative phase of zero corresponds to the situation when both of these events occur simultaneously.

**Fig 2:**
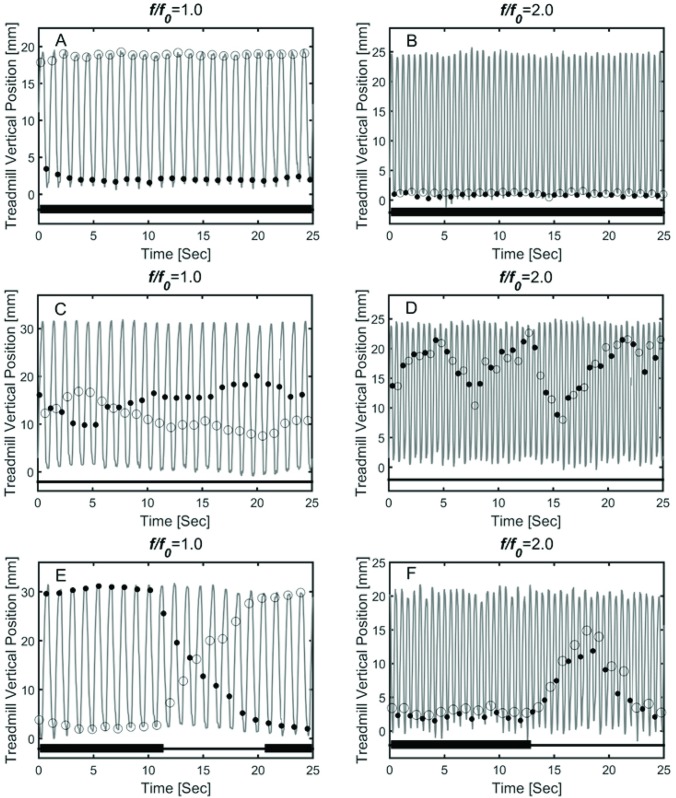
Observed patterns of consecutive heel strike events plotted over the vertical position of the treadmill surface. These examples were obtained from different subjects and different conditions of platform oscillation: A (amp = 2cm, *f* = *f*_*0*_), B (amp = 2.5cm, *f* = 2×*f*_*0*_), C (amp = 3cm, *f* = *f*_*0*_), D (amp = 2.5cm, *f* = 2×*f*_*0*_), E (amp = 3cm, *f* = *f*_*0*_), F (amp = 2cm, *f* = 2×*f*_*0*_). Black dots represent heel strike for the left foot, open circles represent heel strike for the right foot. Periods of either 1:1 or 2:1 phase locking are indicated with a bold line at the bottom of each panel.

The phase locked patterns were described as *n*:*m*, where *n* is the number of cycles of the platform vertical oscillation and *m* is the number of strides (Fig 2). The 1:1 and 2:1 phase locked patterns were identified as those where ϕ, the phase of the heel strike related to the platform oscillation was either ≈ 0° or 180° for 1:1, or some integer multiple of 90° for 2:1. It is important to note that if there is no interaction between the walker and the platform oscillation, then the initial ϕ will be maintained subject to the influence of stochastic fluctuations with f≈f_0_ (Fig 2C). When f≠f_0_ more complex patterns are possible (Fig 2D). These instances of passive interaction between the platform and walker were distinguished from the phase locked patterns by the greater variance in ϕ (variance 5.2–5.8° for phase locked vs 22.2–53.4° when passively coordinated).

### Laminar Phases

Subjects typically phase locked to the platform oscillation for only portions of the 2 min walking trial. We refer to the time interval over which the gait is 1:1 or 2:1 phase locked as, respectively, a *1*:*1 laminar phase* and a *2*:*1 laminar phase*. The lengths of the laminar phases collected for all subjects who performed for a given experimental condition were used to determine the survival function for the laminar phases [24–26]. The survival function, P(t_pl_ > t), was determined by measuring the fraction of trials for which the length of the laminar phase, t_pl_, lasted longer than each successive time point in the trial, t. The data were fit to the Weibull survival function,

$$\text{P}\left(t_{pl}>t\right)\approx exp\left(-\lambda t^{\beta }\right),$$ (3)

where λ and β are positive constants, using programs written in MATLAB. The mean length of the laminar phase is equal to *λ*^−1/*β*^ Г(1 + 1/*β*) where Г refers to the gamma function. The arithmetic mean of laminar phases were also calculated directly from the data for comparison.

### Platform Oscillation Amplitude

The effect of the amplitude of the platform oscillation on gait synchronization was measured for the remaining 5 subjects (age 27.3±6.7 years, mass 66.5±2.6 kg, height 1.6±0.1 m, leg length 76.6±6.0 cm). These subjects performed several walking trials over multiple data collection sessions (a total of 75 trials over 1–3 sessions). As above, the treadmill speed was kept at 4.02 km/h and each session included a 2 min warm up walk followed by a 2 min walk to determine *f*_*0*_. Subjects were then exposed to several different combinations of A and *f* in random order (*f/f*_*0*_ ranging from 0.5 to 2.5 and *A* ranging from 1cm to 6cm). The range of A is comparable to the range of vertical displacements of the center of mass during normal walking on a level surface [27].

### Stride Kinematics

Stride kinematics were calculated using sagittal plane trajectories of the marker placed on the right malleolus. These trajectories were first smoothed by subtracting the vertical treadmill motion artifact from the vertical position of the ankle. Averages and standard deviations were then calculated for stride length, stride height, and stride duration during each trial. Separate repeated measures ANOVA were calculated for each variable. Post hoc analysis involved separate paired t-tests to determine where significant differences lay. The Bonferroni correction was used to adjust for multiple comparisons, with a family-wise alpha level of 0.05.

## Results

Fig 2 shows 6 examples of the relationship between the phase of heel strike and the treadmill oscillation for multiple values of A. The phase locking patterns for VOTW exhibit three types of behaviors. First, Fig 2A and 2B show examples where the phase of heel strike with respect to the treadmill oscillation is constant for many strides. Second, the ϕ can vary in a more complex manner that suggests a passive interaction between the platform oscillation and gait (Fig 2C and 2D). We interpreted these intervals when ϕ varied as instances when the gait cycle and platform oscillation were completely uncoupled. Finally, Fig 2E and 2F show that the transitions between the laminar phases and the intervals of uncoupled dynamics occur abruptly.

**Fig 3:**
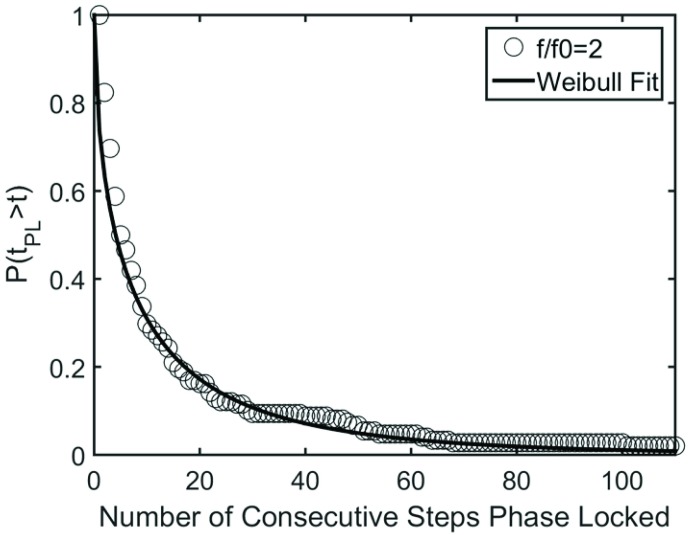
Example of data fit to Weibull survival function *exp*(−*λt*^*β*^). Data are aggregated across 26 subjects (*f/f*_*0*_ = 2.0, Amp = 2.5cm). Open circles represent the percentage of trials that survive at each time point; the solid line represents the Weibull curve with λ = 0.305 and β = 0.585.

**Fig 4:**
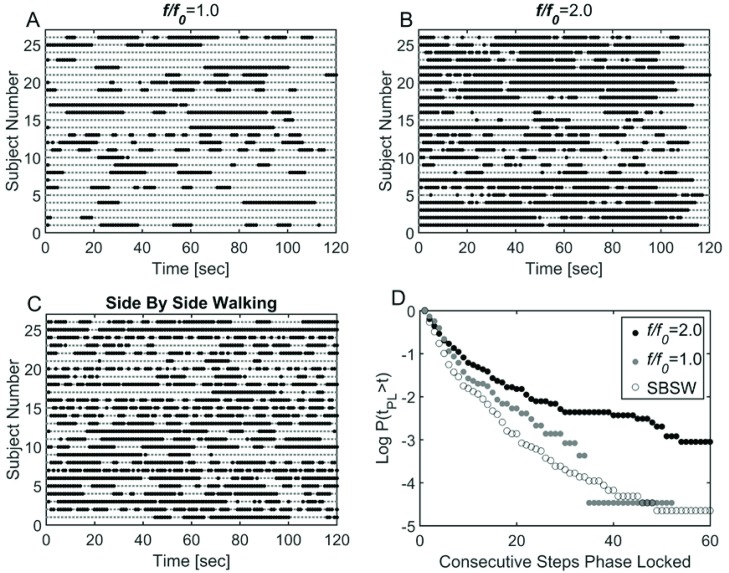
Laminar phase intervals for oscillating treadmill condition at 1:1 (A), 2:1 (B), and during side by side walking (C). Survival curves for each condition are also provided (D).

Taken together the observations in Fig 2 indicate that VOTW exhibits intermittent phase locking, a phenomenon widely observed in neural [28–30] and physiological [31] dynamical systems. Fig 3 provides an example of the Weibull function fit to this intermittent phase locking, and Fig 4 depicts schematically the intermittent nature of the 1:1 (Fig 4A) and 2:1 (Fig 4B) laminar phases for the entire walking trails for all 26 subjects. In general the length of the 1:1 and 2:1 laminar phases (solid bars) was shorter than the length of the walking trial (Fig 4A and 4B). However, the duration of the 2:1 laminar phases tended to be longer than the 1:1 laminar phases. The intermittent nature of the 1:1 laminar phases during SBSW [21,32] is shown in Fig 4C. Fig 4D compares the survival function, P(t_pl_>t), for VOTW and SBSW; the mean length of the laminar phases, λ, and β are summarized in Table 1. It is clear that the laminar phases are longer for VOTW than SBSW. Moreover, for VOTW the 2:1 laminar phases typically last much longer than the VOTW 1:1 laminar phases.

**Table 1:** Laminar phase statistics.

	*f/f*_*0*_	Phase Locked [% of Trial]	λ	β	Laminar Phase Length	
					Weibull	Arithmetic
**SBSW**	1.00	59.2±17.4	0.335	0.75	5.175	7.387
**Stride -5%**	0.95	34.8±9.9	0.435	1.02	2.2432	4.1233
**Stride Matched**	1.00	27.2±19.5	0.2	0.865	6.9186	8.8161
**Stride +5%**	1.05	30.7±13.3	0.245	1.14	3.2767	4.8771
**Step -20%**	1.80	63.5±13.4	1.13	0.635	1.1574	3.1287
**Step -10%**	1.90	58.4±24.5	0.595	0.695	2.6903	4.8588
**Step Matched**	2.00	70.6±24.1	0.305	0.585	11.8479	13.5068
**Step +10%**	2.10	75.7±18.4	0.3	0.8	5.1030	8.5296
**Step +20%**	2.20	78.6±8.3	0.68	0.855	1.7019	3.448

**Fig 5:**
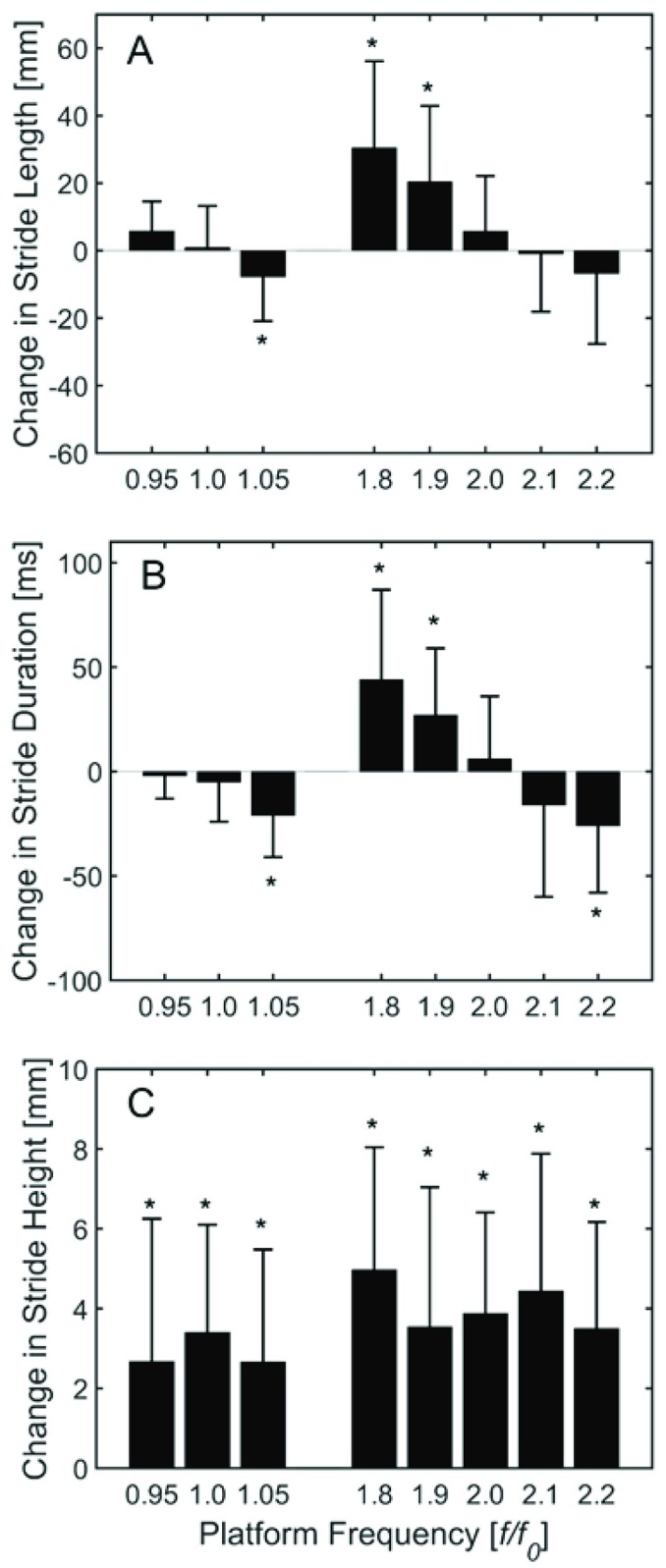
Mean changes in stride length (A), stride duration (B) and stride height (C) as a result of changes to platform oscillation frequency. *denotes significant difference from normal walking condition with no oscillation (p<0.05).

The effects of platform oscillation on gait kinematics and the survival of the laminar phases depends on *f* (Fig 5). There were no significant effects of *f* on stride duration and length when *f* was equal to 0.95 × *f*_*0*_, *f*_*0*_, 2.0 × *f*_*0*_ and 2.1 × *f*_*0*_. However, when *f* was equal to 1.05 × *f*_*0*_ 1.8 × *f*_*0*_, and 1.9 × *f*_*0*_ the subjects made significant changes in stride length and duration. When the stride height was adjusted for treadmill motion it was significantly affected by all values of *f*.

**Fig 6:**
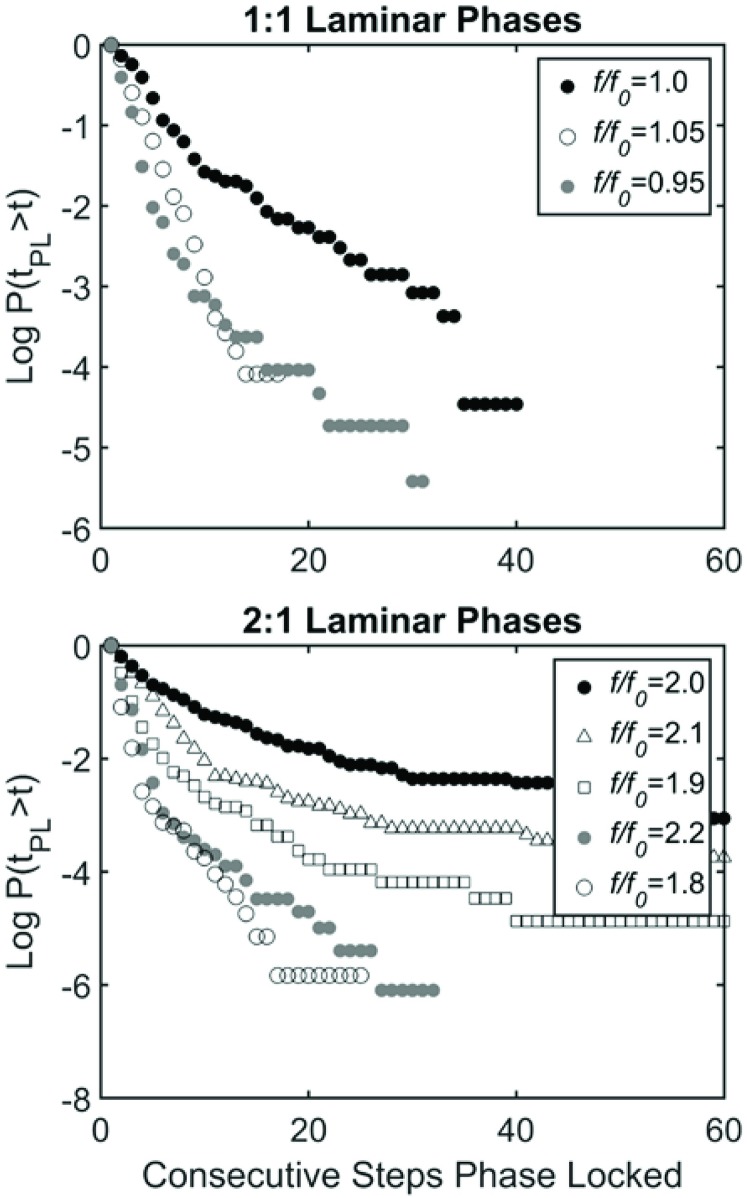
Survival curves for treadmill frequencies in the neighborhood of *f/f*_*0*_ = 1 (1:1 Laminar Phases, top) and *f/f*_*0*_ = 2 (2:1 Laminar Phases, bottom). Curves are truncated to 60 steps for ease of comparison.

Fig 6 and Table 1 compare P(t_pl_ > t) obtained for different *f* when A = 2.5cm. A 10% change in *f/f*_*0*_ produces a slightly larger change in the survival of the 2:1 laminar phases (Fig 6b) when compared to a 5% change in *f/f*_*0*_ in the 1:1 laminar phases (Fig 6a). Thus, it appears as though the 2:1 laminar phases when *f* = 2 × *f*_*0*_ appear to be less sensitive to changes in *f* than the 1:1 laminar phases when *f* = *f*_*0*_. Table 1 summarizes the Weibull parameters for a variety of phase locking behaviors.

**Fig 7:**
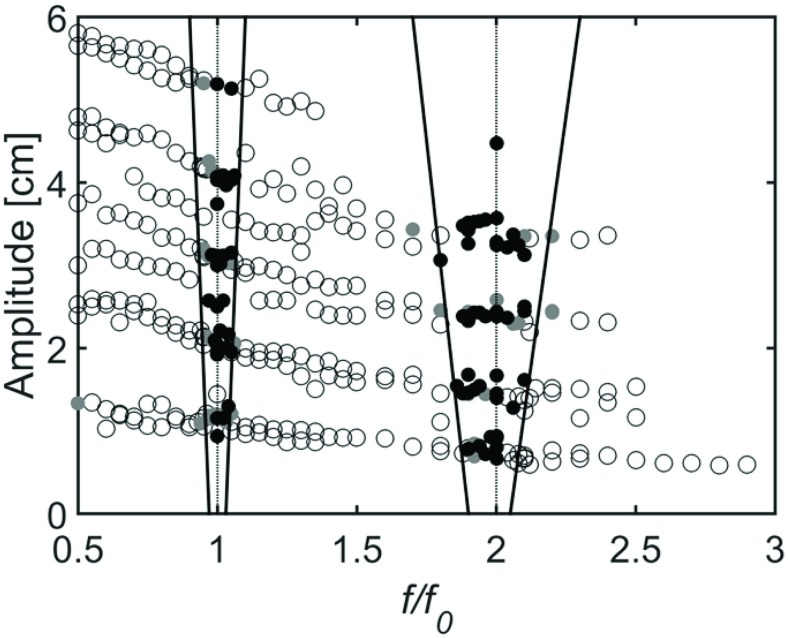
Arnold tongue diagram for synchronization between 5 walking subjects and vertical oscillation of the walking surface. Each dot represents a single 1 minute trial. The presence of laminar phases are represented by Open circles: few laminar phases, Grey dots: moderate laminar phasing, Black dots: consistent laminar phasing. Solid lines denote estimates of regions of stable synchronization (i.e. “tongues”).

Intermittent phase locking is expected to occur when the coupling strength between the two oscillators is moderate. In VOTW, the coupling strength is anticipated to be proportional to A. Fig 7 summarizes the effects of *f* and *A* on gait synchronization in the form of an Arnold tongue diagram [33,34]. We have grouped the combinations of A and *f/f*_*0*_ which produced 1:1 or 2:1 laminar phases together into an Arnold tongue. As can be seen there is a slight tendency for the percentage of the walking trial which is phase locked to increase as *A* increases (more black dots). Further, the relative width of each “tongue” increases with increasing A, though this increase is relatively small. Additional analysis of these data suggest that treadmill frequency had a much greater effect on phase locking than changes in amplitude. For example, using simple linear regression it was determined that a change in amplitude of 10mm resulted in a change in phase locking percentage of approximately 1.6% across a 60 second trial. Further, relative phase angle at heel strike between the subject and platform was moderately correlated with the platform’s deviation from preferred stride frequency (r = 0.61) but not with platform amplitude (r = -0.05). These results suggest that variations in amplitude that occurred as a result of power limitations of the pneumatic actuators (i.e. due to heavier subjects) had a minimal effect on the analyses performed here.

## Discussion

Our observations demonstrate that VOTW results in significantly greater unintentional synchronization than occurs during SBSW. When *f/f*_*0*_ = 1.0 or *f/f*_*0*_ = 2, the increased synchronization occurs without significant changes in stride length or stride duration. These observations suggest that input signals that activate multiple sensory pathways, including haptic and vestibular feedback, result in a greater amount of unintentional synchronization. The Arnold tongue diagram depicts a broader “tongue” for synchronization at *f/f*_*0*_ = 2.0 suggesting that the 2:1 laminar phases are more stable that the 1:1 laminar phases for human gait. In the case of *n*:*m* phase locking the width of the Arnold tongue for fixed amplitude stimulation is known to satisfy geometric scaling laws in the case of vanishing amplitude [35]. These results imply that in an experimental setup such as ours only entrainment patterns with small values of *n* and *m* will be observed.

It is surprising that the survival of the 2:1 laminar phases is longer than that of the 1:1 laminar phases. Typically in neural systems, 1:1 phase locking patterns are regarded to be the most robust since, for example, they persist even in the limit of vanishingly small modulation amplitudes [36]. However, little is known about the effects of a vertically moving walking surface on gait kinematics [37]. For the current data, a difference between the 1:1 and 2:1 laminar phases is that in the former the heel strikes occur at different platform heights, whereas in the latter the heel strikes all occur at the same platform height. Since the energetic costs of walking are related to vertical displacements of the center of mass [37,38], it is possible that the 2:1 laminar phases survive longer because less work is required for their maintenance. This observation may also explain why for some uncoupled VOTW, the phase of the heel strikes tend to cluster around the mid-point of the platform oscillation (Fig 2C).

The lengths of the 1:1 and 2:1 laminar phases are sensitive to changes in *f*. Deviations from the stride-matched and step-matched frequencies resulted in an increase in β and a decrease in the length of the laminar phase. Presumably these changes reflect the response of the haptic and vestibular feedback loops to the increased vertical velocity and acceleration of the movements of the walking surface. Additional study of laminar phase characteristics in response to other combinations of frequency and amplitude VOTW, or while attenuating certain aspects of sensory feedback, may provide clarification on the role of the vestibular and visual systems in synchronization of gait to platform motion.

The Weibull function provided an approximation of the survival of laminar phases during intermittent synchronization. The correlation between the Weibull prediction and the arithmetic mean was very high (R^2^ = 0.96) and the average fit of the Weibull function to the survival data generated a relatively low RMS error (mean error = 0.012 strides). However, the mean length of the laminar phases for all conditions was larger than that predicted (Table 1). This observation is not entirely unexpected since for gait synchronization the tendency of a laminar phase to fail is opposed by the stability, albeit weak, of the phase locked state. Consequently the longer the laminar phase persists, the more likely it is to persist even longer [24,25]. Since this effect is not taken into account by eq 1, the predicted mean length of the laminar phases will be less than the measured one.

The intermittent nature of human gait synchronization has received little attention. Intermittent phase locking between two oscillators arises when the coupling strength is too weak to maintain synchronization. Many different mechanisms have been identified to produce intermittent synchrony [28–30]. Some of these mechanisms can be distinguished by careful attention to the statistical properties of the laminar phases, in particular by the dependence of the mean length of the laminar of phase on a critical parameter or by the nature of the distribution of laminar phase durations. For the current analysis, intermittent synchronization was investigated within the context of 1:1 or 2:1 phase locking patterns, but other, more complex behaviors may also be present (3:2 or 4:3, for example) and can be evaluated in future experiments.

Vertical oscillation may also be useful for altering sagittal plane kinematics during gait. In particular, treadmill oscillations at frequencies below participants’ preferred stride frequency led to significant increases in both stride length and stride duration, while frequencies above their preferred stride frequency had the opposite effect. This behavior might be explained mechanically: if participants are attempting to synchronize, it is reasonable to expect that they will adjust their stride duration to match the platform oscillation frequency. Since walking speed is related to both stride length and duration, a change in stride duration will require a change in stride length to maintain equilibrium on the treadmill belt (assuming constant treadmill speed). On the other hand, stride height was also significantly altered, though rather than demonstrating a modulation across treadmill frequencies it was increased for all walking conditions. This may reflect a compensation for uncertainty in the vertical motion of the walking surface as it moves in a pattern that was presumably unknown to participants.

Intermittent phase locking may facilitate high adaptability of biological systems as they react to different environmental impacts [30]. An advantage of the VOTW over the SBSW paradigm is that it permits selective manipulation of the properties of the oscillatory treadmill. This makes it possible to influence several aspects of a person’s gait in a functional way. Thus these findings may have implications for the use of sensory motor synchronization for rehabilitation.
